# Oxygenation and ventilation during prolonged experimental cardiopulmonary resuscitation with either continuous or 30:2 compression-to-ventilation ratios together with 10 cmH_2_0 positive end-expiratory pressure

**DOI:** 10.1186/s40635-024-00620-z

**Published:** 2024-04-12

**Authors:** Jukka Kopra, Erik Litonius, Pirkka T. Pekkarinen, Merja Laitinen, Juho A. Heinonen, Luca Fontanelli, Markus B. Skrifvars

**Affiliations:** 1 Department of Emergency Care and Services, University of Helsinki and Helsinki University Hospital, Helsinki, Finland; 2 Division of Anaesthesiology, Department of Anaesthesiology, Intensive Care and Pain Medicine, University of Helsinki and Helsinki University Hospital, Helsinki, Finland; 3 Division of Intensive Care, Department of Anaesthesiology, Intensive Care and Pain Medicine, University of Helsinki and Helsinki University Hospital, Helsinki, Finland; 4 VetCT Teleconsulting-Teleradiology Small Animal Team, Helsinki, Finland; 5 Centre for Prehospital Emergency Care and Emergency Medicine, Päijät-Häme Central Hospital, Lahti, Finland; 6 Department of Clinical-Surgical, Diagnostic and Paediatric Sciences, Unit of Anaesthesia and Intensive Care, University of Pavia, Pavia, Italy

**Keywords:** Cardiac arrest, Cardiopulmonary resuscitation, Ventilation, Hypoxaemia, Arterial oxygen pressure, Mechanical chest compression, Electrical impedance tomography, Computed tomography

## Abstract

**Background:**

In refractory out-of-hospital cardiac arrest, the patient is commonly transported to hospital with mechanical continuous chest compressions (CCC). Limited data are available on the optimal ventilation strategy. Accordingly, we compared arterial oxygenation and haemodynamics during manual asynchronous continuous ventilation and compressions with a 30:2 compression-to-ventilation ratio together with the use of 10 cmH_2_O positive end-expiratory pressure (PEEP).

**Methods:**

Intubated and anaesthetized landrace pigs with electrically induced ventricular fibrillation were left untreated for 5 min (*n* = 31, weight ca. 55 kg), after which they were randomized to either the CCC group or the 30:2 group with the the LUCAS® 2 piston device and bag-valve ventilation with 100% oxygen targeting a tidal volume of 8 ml/kg with a PEEP of 10 cmH_2_O for 35 min. Arterial blood samples were analysed every 5 min, vital signs, near-infrared spectroscopy and electrical impedance tomography (EIT) were measured continuously, and post-mortem CT scans of the lungs were obtained.

**Results:**

The arterial blood values (median + interquartile range) at the 30-min time point were as follows: PaO_2_: 180 (86–302) mmHg for the 30:2 group; 70 (49–358) mmHg for the CCC group; PaCO_2_: 41 (29–53) mmHg for the 30:2 group; 44 (21–67) mmHg for the CCC group; and lactate: 12.8 (10.4–15.5) mmol/l for the 30:2 group; 14.7 (11.8–16.1) mmol/l for the CCC group. The differences were not statistically significant. In linear mixed models, there were no significant differences between the groups. The mean arterial pressures from the femoral artery, end-tidal CO_2_, distributions of ventilation from EIT and mean aeration of lung tissue in post-mortem CTs were similar between the groups. Eight pneumothoraces occurred in the CCC group and 2 in the 30:2 group, a statistically significant difference (*p* = 0.04).

**Conclusions:**

The 30:2 and CCC protocols with a PEEP of 10 cmH_2_O resulted in similar gas exchange and vital sign outcomes in an experimental model of prolonged cardiac arrest with mechanical compressions, but the CCC protocol resulted in more post-mortem pneumothoraces.

**Supplementary Information:**

The online version contains supplementary material available at 10.1186/s40635-024-00620-z.

## Background

International cardiopulmonary resuscitation (CPR) guidelines recommend continuous ventilations with an advanced airway at a rate of 10–12 breaths per minute. When using a supraglottic airway device and there is a significant leak, a 30:2 ratio of compressions to ventilations is preferred [1, 2]. In cases of out-of-hospital cardiac arrest (OHCA), where return of spontaneous circulation (ROSC) is not achieved on scene, the patient is commonly transported to hospital with ongoing CPR. In these situations, mechanical chest compression devices are recommended to ensure rescuer safety and provide high-quality compressions [3, 4]. Nonetheless, even with a secured airway and the use of an FiO_2_ of 1.0, many patients arriving at hospital have severe hypoxia, hypercarbia and acidosis on blood gas analysis [5–8]. This may predispose patients to brain injury and life-threatening lung and immunological reactions in those who achieve ROSC or in whom ECPR is initiated.

Even though mechanical compression devices provide superior perfusing pressures in comparison to manual compressions [9], their effect on ventilation strategies is less known [10, 11]. During patient transport, when providing manual chest compressions is difficult or impossible, using a mechanical chest compression device has been shown to improve compression quality [3, 4]. But using a mechanical compression device could impair ventilatory performance because of dynamic air trapping [12] and atelectasis formation. Previously, we compared the 30:2 protocol with continuous chest compressions (CCC) in an experimental CPR model of prolonged resuscitation [13] and found no statistically significant differences between the study groups.

One option to improve gas exchange is the use of positive end-expiratory pressure (PEEP) in delivering ventilation. Experimental studies have suggested improved oxygenation with PEEP [14–16] but it has not been conclusively studied in the setting of prolonged CPR using mechanical chest compressions. In the current study, we compared oxygenation and ventilation between continuous compressions and a 30:2 compression-to-ventilation ratio, together with the use of 10 cmH_2_O of PEEP. We hypothesized that a 30:2 compressions to ventilation ratio would provide higher better oxygenation than continuous compressions and ventilation. The primary endpoint of this study was the levels of oxygen over time between the two groups. The secondary endpoints included arterial levels of carbon dioxide and lactate, brain oxygenation analysed with NIRS over time, the distribution of ventilation with electrical impedance tomography (EIT) recordings and post-mortem computed tomography (CT) scans.

## Materials and methods

We conducted this experimental animal study on healthy landrace pigs (*n* = 31, both sexes) at the Laboratory Animal Centre, Large Animals Unit at the Viikki Campus of the University of Helsinki between May 2021 and December 2022. The study was approved by the Finnish National Animal Experiment Board (ESAVI/ 4121/2021). The study is reported in adherence to the ARRIVE guidelines [17], and a checklist is included in Additional file 1.

### Preparation and monitoring

The fasted animals were premedicated with intramuscular injections of medetomidine (9–10 mg) and racemic ketamine (450–500 mg). An IV line was inserted into an ear vein, and anaesthesia was induced with IV propofol (dose 1–1.5 mg/kg) and fentanyl (3–4 mcg/kg), after which endotracheal intubation was performed (internal diameter 6.0–7.0 mm). Mechanical ventilation was started (Servo Ventilator 900C, Siemens-Elema, Solna, Sweden) with 21% oxygen (O_2_) and an end-tidal carbon dioxide (EtCO_2_) target of 5%. The internal jugular vein and femoral artery were cannulated (arrow, size 8.5 Fr. 16 cm, Teleflex Medical Europe Ltd, Westmeath, Ireland and Avanti+, size 6F, length 11 cm, Cordis, Tipperary, Ireland, respectively), and baseline blood samples were taken. A temporary pacemaker wire was inserted next, and the right ventricular location was confirmed with the achievement of ventricular pacing (Medtronic 5348 Single Chamber Temporary Pacemaker, Soma Tech INTL, Bloomfield, CT, USA).

Arterial blood samples were analysed with a point-of-care device (i-STAT System, Abbott Laboratories, Princeton, NJ, USA), and haemodynamic and respiratory variables, including spirometry, were monitored using a Datex-Ohmeda AS/3 monitor with a M-COVX gas module (GE Healthcare, Helsinki, Finland) and recorded using data collection software (iCentral® and S/5 Collect®, GE Healthcare, Helsinki, Finland). A rectal temperature probe was inserted, and a normal temperature of 38 °C–39 °C was targeted with the use of warm blankets when necessary. Cerebral oximetry was performed with NIRS (INVOS TM5100C Cerebral Oximeter, Somanetics Inc., Troy, MI, USA) with one sensor in the forehead and another on the belly as a control. The EIT belt was set around the chest just caudal to the compression device piston. The hair under the belt was shaved off, and the skin was cleaned with 70% ethanol to ensure the best possible conductivity. The EIT data were recorded with a Dräger Pulmovista® 500 (Drägerwerk AG & Co., Lübeck, Germany) and analysed with a Dräger SW EITdiag V1.6 (Drägerwerk AG & Co., Lübeck, Germany).

### Experimental procedures

**Fig. 1:**
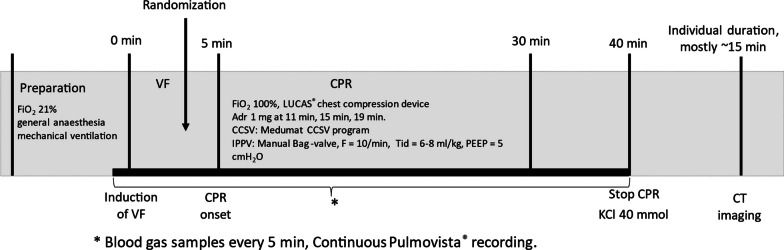
Timeline of the experimental protocol. *Adr* adrenaline, *FiO*_*2*_ fraction of inhaled oxygen, *VF* ventricular fibrillation, *CPR* cardiopulmonary resuscitation, *KCl* potassium chloride, *CT* computed tomography

Figure 1 presents the experiment timeline. After VF was induced with a 9 V direct current, ventilation and anaesthesia were ceased and the pacing wire was removed. Randomization into the study groups of either CCC or the 30:2 protocol was performed with sealed opaque envelopes during the 5-min hands-off period. In the meantime, a Lucas 2® compression device (Jolife AB, part of Stryker, Lund, Sweden) was set up and the position of the pig was stabilized with a vacuum mattress and a handful of towels within the Lucas 2® arch. Manual bag-valve ventilation (LAERDAL Silicone Resuscitator, Laerdal Medical, Stavanger, Norway) was performed with 100% oxygen, either with a frequency of around 10 min^−1^ in the CCC group or twice during the compression pause in the 30:2 group, targeting an approximate tidal volume of 500 ml in both groups. The PEEP of 10 cmH_2_O was adjusted with an Ambu® PEEP valve (Ambu A/S, Ballerup, Denmark). Adrenaline was administered as a 1 mg IV dose at 11-, 15- and 19-min time points. A 20-s recording break from compressions for the EIT was held at the 30-min time point with continuous ventilation in both groups for the duration. Arterial blood samples were collected at 5-min intervals, and a venous sample was also taken at the 30-min time point. Monitoring, NIRS and EIT were recorded continuously through the experiment. The animals were killed with a 40 mmol dose of potassium chloride at the 40-min time point. Simultaneously, the intubation tube was clamped after insufflating the lungs with one full manual ventilation. The post-mortem CT scans were collected in the prone position approximately 15 min after the cessation of CPR.

### Data processing

**Fig. 2:**
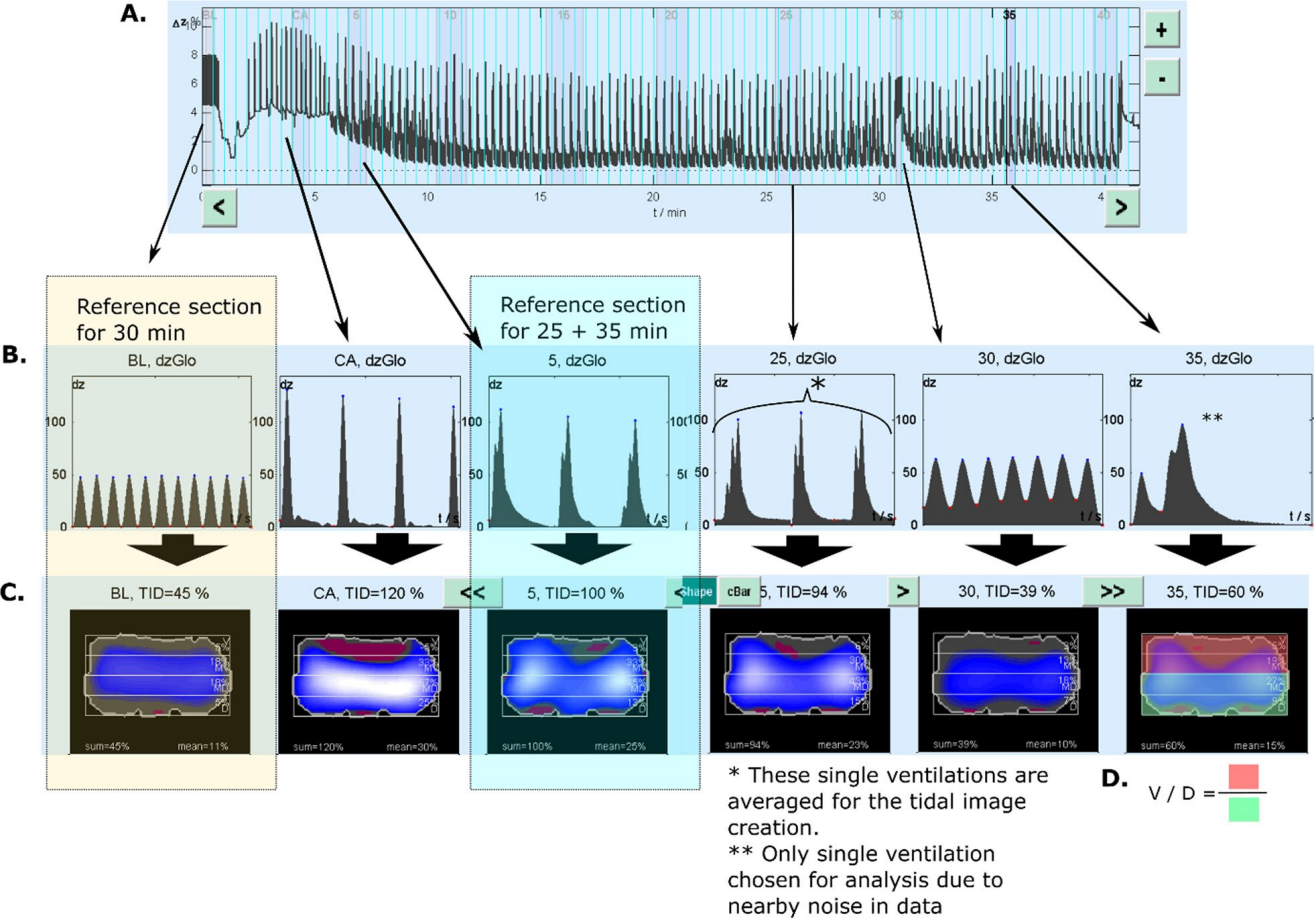
Schematic illustration of the EIT analysis work chart. **A** The raw dzGlo curve. **B** The reconstructed sections after filtering. **C** Visual slices created with the automated analysis scheme from filtered sections. **D** Visual explanation of the V/D index. *BL* baseline, *dzGlo* global impedance change, *TID* tidal impedance change, *CA* cardiac arrest, *V/D* ventral to dorsal

An illustration of the EIT analysis is provided in Fig. 2. Sections of interest were chosen from the raw recording (baseline, cardiac arrest and 5-min intervals throughout the CPR). The reconstruction settings are provided in Additional file 2. The reconstructed sections were analysed using automated methods provided by the analysis software to create visual slices and quantified data. The global tidal impedance change (dzGlo) was referenced to the 5-min section, except for the 30-min section, which was referenced to the baseline section. The ventral to dorsal (V/D) distribution indices were computed, as illustrated in Fig. 2. A V/D value of 1 represents an equal distribution of ventilation, and a value of 0 means that all tidal change happens dorsally.

The CT scans were evaluated by a veterinary radiologist (ML) blinded to the intervention group with Osirix MD version 12.5.0 (VetCT, Cambridge, UK). The Hounsfield unit (HU) values were measured from 10 lung slices chosen as described by Reske et al. [18]. The first slice was chosen from the most cranial aspect of the lungs, where both the left and right cranial lobes are visible on the same transverse slice. The most caudal slice was chosen similarly where there was still enough lung on both hemithoraces for segmentation into 2–3 segments, but where the accessory lobe was no longer visible. Eight evenly spaced slices were chosen from between them.

An illustrative figure of HU measurements is provided in Additional file 3. The lung parenchyma was manually delineated separately for the hemithoraces and accessory lobes. The measured mean HU values for each hemithorax, normalized to the area of the delineation, were used to calculate the mean HU of the overall lung. The hemithoraces were also divided into 2–3 ventro-dorsal segments of equal height, and representative HU values of these segments were averaged for the dorsal, middle and ventral HU values without normalization to area. Subjects with more than a mild pneumothorax were excluded from the analysis because of the dislocated anatomy.

### Statistical analysis and sample size

For the sake of clarity, all variables are reported as medians and interquartile ranges (IQRs), since most variables violated the assumptions of parametric testing. Single comparisons were tested with the Mann–Whitney *U* test for statistical significance. Comparisons with multiple time point measurements were tested with a linear mixed-effects model and a heterogeneous Toeplitz covariance matrix. The effects of the interventional group, time and interaction between time and the intervention group were included in the model. The values over time were plotted as medians and IQRs.

The sample size was estimated based on arterial blood gas data from Kim et al. [19]. According to their data, a sample size of 30 divided into two equal groups would be sufficient to detect a 20 mmHg difference in PaO_2_ with a statistical power of 0.80 and an alpha of 0.05.

As an exploratory analysis, we also compared the results of the PEEP zero pigs from our previous study [13] to the PEEP 10 pigs of the current study.

## Results

We randomized 31 animals (30:2 group: *n* = 16; CCC group: *n* = 15). Persisting agonal breathing until the 30-min time point was witnessed in 2 pigs in the 30:2 group and in 6 pigs in the CCC group. We encountered no cases of spontaneous ROSC. Most animals deteriorated to ASY soon after the perfusing pressures withered.

There were no significant differences in the baseline characteristics (Table 1). The FiO_2_, peak airway pressure or compliance levels during advanced life support (ALS) were similar between the groups (Table 2).

**Table 1:** Baseline characteristics of the intervention groups

Prearrest	30:2 CPR Median (IQR)	Continuous CPR Median (IQR)	*p* value
Weight	55 (45–59)	57 (50–58)	0.77
Heart rate (bpm)	122 (106–131)	113 (94–137)	0.94
FiO_2_ (%)	21 (21–21)	21 (21–21)	0.52
Peak airway pressure (cmH_2_O)	27 (25–28)	25 (23–28)	0.74
Tidal volume (ml)	380 (350–410)	420 (370–460)	0.23
Compliance (ml/cmH_2_O)	30.1 (26.6–39.6)	35.0 (28.1–44.6)	0.60
pH	7.55 (7.53–7.56)	7.54 (7.53–7.56)	0.83

*CPR* cardiopulmonary resuscitation, *IQR* interquartile range, *bpm* beats per minute, *FiO*_*2*_ fraction of inspired oxygen

**Table 2:** Vital signs and physiologic variables during cardiopulmonary resuscitation

Cardiac arrest phase	30:2 CPR Median (IQR)	Continuous CPR Median (IQR)	*p*-value
Ventilation rate (min^−1^)	5 (5–7)	9 (9–10)	> 0.001
FiO_2_ (%)	94 (93–95)	94 (94–95)	0.054
Peak pressure (cmH_2_O)	35 (31–41)	37 (31–44)	0.18
Compliance (ml/cmH_2_O)	21.5 (18.5–27.4)	22.6 (17.3–28.6)	0.93
Measured PEEP (cmH_2_O)	9.3 (6.3 – 10.0)	8.5 (6.6 – 10.9)	0.54

*CPR* cardiopulmonary resuscitation, *IQR* interquartile range, *FiO*_*2*_ fraction of inspired oxygen

### Oxygen, carbon dioxide, lactate, mean arterial pressure, end-tidal carbon dioxide and near-infrared spectroscopy

**Fig. 3:**
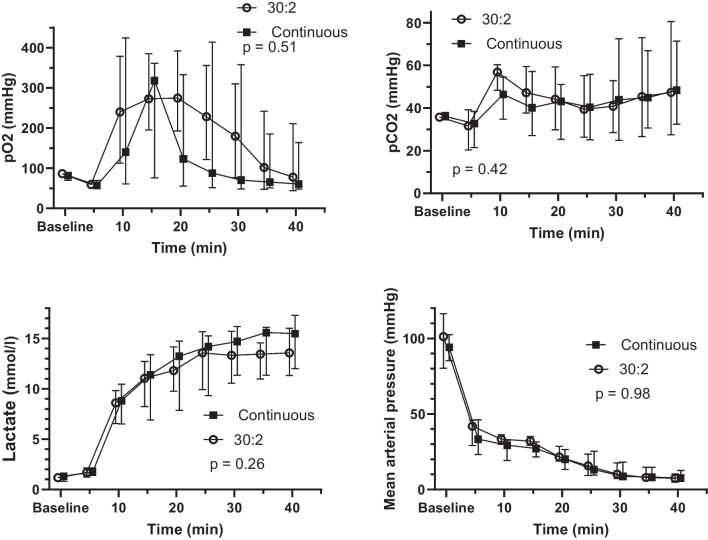
Arterial oxygen pressures (PaO_2_), carbon dioxide pressures (PaCO_2_), lactate levels and mean arterial pressures during experimental cardiopulmonary resuscitation shown as medians and interquartile ranges. The *p* value is given for a linear mixed model between the groups. *CCC* continuous chest compressions

The arterial blood oxygen, carbon dioxide, lactate and mean arterial pressure (MAP) results are shown in Fig. 3. There were no statistically significant differences between the groups in PaO_2_ (*p* = 0.51), PaCO_2_ (*p* = 0.42), lactate (*p* = 0.26) and MAP (*p* = 0.98) or in the time and group interaction terms in the PaO_2_ (*p* = 0.80), PaCO_2_ (*p* = 0.15), lactate (*p* = 0.80) and MAP levels (*p* = 0.17).

**Fig. 4:**
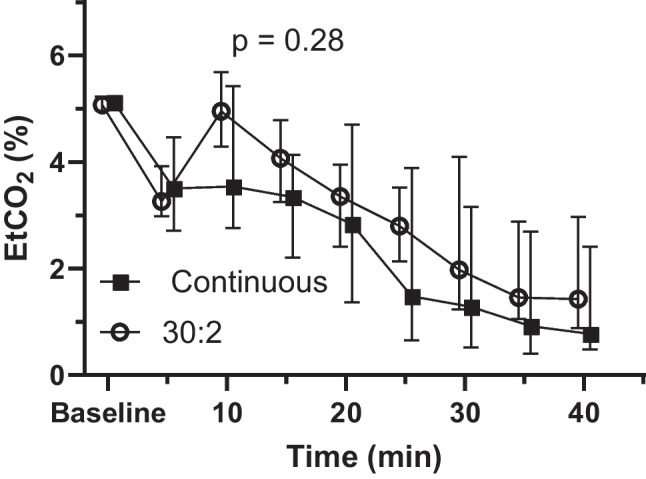
EtCO_2_ levels of the 30:2 and CCC groups during experimental cardiopulmonary resuscitation shown as medians and interquartile ranges. The *p *value is given for a linear mixed model between the groups. *EtCO*_*2*_ end-tidal carbon dioxide, *CCC* continuous chest compressions

The EtCO_2_ levels (Fig. 4) were similar between the groups (*p* = 0.28). The *p* value for the group and time interaction was 0.028.

**Fig. 5:**
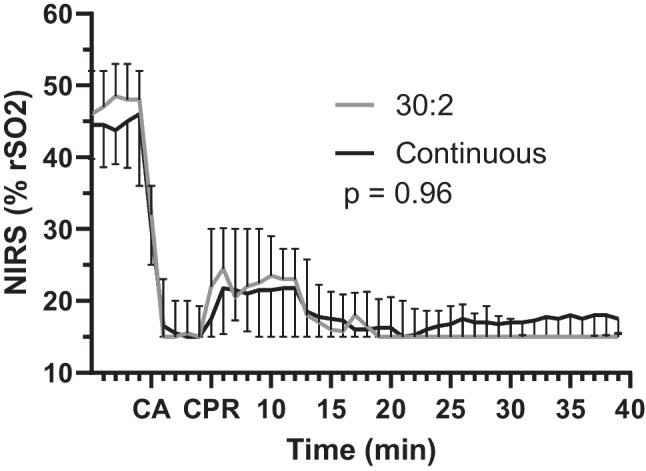
NIRS values during experimental cardiopulmonary resuscitation shown as medians and interquartile ranges. The *p *value is given for a linear mixed model between the groups. *NIRS* near-infrared spectroscopy, *rSO*_*2*_ regional oxygen saturation index, *CA* cardiac arrest, *CCC* continuous chest compressions

The cerebral NIRS values are presented in Fig. 5. One subject from the 30:2 group was removed from the analysis because of clearly abnormal measurement values, suggesting technical failure in the measurement. There was no significant difference between the groups (*p* = 0.96) or between the time and group interaction terms (*p* = 0.36).

### Electrical impedance tomography and computed tomography

The main findings from the EIT data are reported in Table 3. One subject had missing data from the baseline measurement in the CCC group. There were no statistically significant differences in the recorded tidal impedance changes between the groups, although there was a nonsignificant trend of the CCC group having more dorsally distributed impedance changes in all the measurements. Figure 2 depicts the analysis method used to obtain the reported values.

**Table 3:** Electrical impedance tomography and computed tomography findings

EIT findings	30:2 group	CCC group	*p* value
	Median (IQR) *n* = 16	Median (IQR) *n* = 15	
Tidal volume distribution, baseline	0.89 (0.83–0.96)	0.94 (0.80–1.1)*	0.40
Tidal impedance change, 25 min (%)	97 (81–115)	100 (90–116)	0.65
Tidal volume distribution, 25 min	0.92 (0.74–1.0)	0.80 (0.70–0.87)	0.11
Tidal impedance change, 30 min (%)*	133 (109–163)	155 (129–172)	0.42
Tidal volume distribution, 30 min*	0.73 (0.64–0.87)	0.67 (0.58–0.78)*	0.34
Tidal impedance change, 35 min (%)	92 (84–123)	102 (86–117)	0.83
Tidal volume distribution, 35 min	0.93 (0.74–0.98)	0.78 (0.75–0.89)	0.19

CT findings after death	*n* = 16/15^#^	*n* = 14/9^#^	
Total lung volume (ml)	1228 (1002–1447)	1336 (836–1643)	0.89
Rib fractures (#)	5 (4–5)	5 (4–6)	0.15
Mean density (HU)	− 652 (− 588 to − 695)	− 688 (− 589 to − 763)	0.31
Ventral lung density (HU)	− 741 (− 678 to − 781)	− 744 (− 719 to − 833)	0.57
Middle lung density (HU)	− 702 (639 to − 751)	− 728 (− 640 to − 799)	0.50
Dorsal lung density (HU)	− 654 (− 542 to − 668)	− 662 (− 532 to − 755)	0.43
Pneumothoraces (mild/moderate/marked)	2/0/0	6/2/2	0.04
No pneumothorax	14	6	

The values are reported as medians and interquartile ranges. The tidal volume distributions were calculated by dividing the impedance change in the ventral region of interest by the impedance change in the dorsal region of interest. The 5-min recording section was used as a reference section for the 25- and 35-min values, whereas the baseline section was used for the 30-min values. Impedance changes are reported as a comparison value for the chosen reference section. *EIT* electrical impedance tomography, *CCC* continuous chest compressions, *HU* Hounsfield unit. *n = 14; ^#^*n* = general CT findings/HU value findings

Regarding the CT data, one pig in the CCC group did not have the planned scan due to logistical problems. There were no statistically significant differences between the groups in the number of rib fractures, mean lung density of the lungs or regional mean densities (V/D regions reported). One pig in the 30:2 group and 5 pigs in the CCC group were excluded from the HU analysis because of deformed lung anatomy due to pneumothoraces.

Table 3 shows the distribution of the different severity classes of pneumothoraces; 8/10 of the pneumothoraces accumulated in the CCC group and the finding reached statistical significance (*p* = 0.04 with Fisher’s exact test.)

### Exploratory analyses

We analysed the non-randomized comparisons of the primary outcomes (PaO_2_, PaCO_2_, lactate, EtCO_2_ and MAP) of our prior experiment [13] to our current experiment, creating groups of PEEP = 0 cmH_2_O and PEEP = 10 cmH_2_O (*n* = 30 and 31, respectively). The results are reported in Additional file 4. The PaO_2_ levels were significantly higher and the PaCO_2_ levels were lower in the PEEP 10 cmH_2_0 group compared to the PEEP 0 cmH_2_O group. Lactate, MAP and EtCO_2_ levels were similar.

We also compared the PaO_2_ levels between the 30:2 groups, the CCC groups and the 30:2 PEEP = 10cmH_2_O with CCC PEEP = 0 cmH_2_O (also in Additional file 4). The PaO_2_ levels were significantly higher in the 30:2 PEEP 10 cmH_2_O group compared to the 30:2 PEEP 0 cmH_2_O, but not compared to the CCC PEEP 0 cmH_2_O. The CCC PEEP 10 and PEEP 0 cmH_2_O groups had similar PaO_2_ levels. The *p* values were obtained with a linear mixed model.

## Discussion

### Key findings

Contrary to our primary hypothesis, we found no significant differences in arterial blood gases or lactate between CCC and a 30:2 CPR ratio when a PEEP of 10 cmH_2_O was used. Regarding the secondary outcomes, the haemodynamic markers and EtCO_2_ levels were similar between the groups, nor were there any significant differences in the distribution of ventilation or mean lung densities. However, there were significantly more pneumothoraces in the CCC group. These findings suggest that when using PEEP, the CCC and 30:2 protocols during prolonged CPR result in comparable oxygenation and ventilation.

### Relationship to previous studies

Limited patient data on intra-arrest ventilation in patients receiving mechanical chest compressions are available. Mechanical compression devices do not appear to improve survival in patients compared to manual compressions [10], and according to experimental data, their use may be linked to ventilation problems and lung injury [12, 20]. Some previous studies have investigated the use of PEEP as a means of improving oxygenation. One trial on the use of PEEP during CPR with pigs was performed in 1980, showing improved ventilation at the cost of reduced carotid blood flow [16]. This early study was performed with 3:1 and 5:1 compression-to-ventilation ratios. Ruemmler et al. [21] compared guideline-recommended CCC with asynchronous ventilation with and without a PEEP of 5 cmH_2_O PEEP in a porcine model. They found no differences in intra-arrest gas exchange or pulmonary shunting, but found less shunting immediately post-ROSC with the use of PEEP and less immunological mRNA activity in brain tissue samples obtained after ROSC. The intra-arrest peak pressures remained lower with PEEP.

Renz et al. [15] studied the effect of PEEP 8 mbar and 16 mbar on both conventional and ultralow tidal volume ventilation protocols in a prolonged experimental model similar to ours. Within the conventional protocols with varying PEEP levels, they found significant improvements in PaO_2_ levels with 8 mbar of PEEP. In addition, they found lower driving pressures and less post-mortem lung tissue sample trauma findings with 8 compared to 0 mbar of PEEP. Interestingly, the PEEP 16 mbar group had quite poor PaO_2_ in their final measurement point of 25 min, possibly suggesting deleterious intrathoracic pressure development, even though this did not influence the MAP values. The measured level of shunting remained reasonably low in the study with all groups. Also, Levenbrown et al. [14] reported rising PaO_2_ with increasing PEEP (0–5–10–15–20 cmH_2_O) and dose-dependent decreases in measured CO (cardiac output) with rising PEEP levels. They found optimal DO_2_ (oxygen delivery to tissue) with a PEEP level of 5 cmH_2_O.

Comparing our primary outcome results to our previous study in the same setting [13], the findings of higher PaCO_2_ and EtCO_2_ levels in the 30:2 protocol were not reproduced in this experiment. The PaO_2_ levels throughout the experiment were significantly higher in the 30:2 group with PEEP than without it (figures and statistics reported in Additional file 4). In the CCC group, paO_2_ was similar with and without PEEP. This points to the possibility of positive effects of PEEP when added to the 30:2 protocol, but not with the CCC protocol, contrary to some previous findings by other research groups. The possible benefit to ventilatory performance in the 30:2 protocol with the added PEEP possibly results from the prevention of atelectasis formation during the compression phase. In future, it could be worthwhile to research the 30:2 protocol with continuous ventilations preserving the short compression breaks for uninterrupted ventilations.

Mälberg et al. [22] recently published a 20-min experimental CPR model comparing CCC and 30:2 with monitoring focussed on airway pressures and tidal volumes and found peak airway pressures rising higher in the CCC group when the tidal volumes were strictly kept similar between the groups. The CO_2_ levels were lower in the arterial samples and expiratory gases and pH were higher in the CCC group, reinforcing the trends found in our previous work.

### Study strengths and limitations

The landrace pig is the most established model animal of CPR, and we used fairly large animals resembling more mature anatomy and physiology. The current study utilized a model of prolonged CPR. The importance of ventilatory performance in ALS is pronounced when the cardiac arrest time exceeds 10 min and rising PaCO_2_ levels start to cause acidosis, which makes the heart more resistant to defibrillations and possibly results in an aggravated insult to brain tissue, derangements of cerebral blood flow and more pronounced general immunological reactions analogous to a trauma-induced DO_2_ debt [23]. Research on ECPR has demonstrated that CPR times with a low-flow state last up to 60 min amongst recruited patients [24], and we believe that our experimental setup modelled this situation with clinical relevance.

We note certain limitations of this experimental study. The variance between our subjects remained high, especially after prolonged CPR (> 15 min). This may relate to factors influencing the efficiency of blood flow, such as small compression point variations [25, 26]. A clinically more relevant experimental model ideally should be extended to as long as 60 min of CPR. In the current model we did however often experience loss of systemic perfusion pressure within the 20–30 min frame based on a drop in EtCO_2_, deterioration of VF to asystole and increasing lactate, hypoxia and hypercarbia contradicting the timeframe expansion.

Also, the occurrence of agonal breaths seemed to affect oxygenation, but these may occur in humans as well. The thoracic anatomy differs markedly from humans and some phenomena seen in this study may not be relevant to patient work. The pig anatomy may predispose it to more trauma from compressions.

We found no significant differences in the peak airway pressures. Ventilations were provided by a member of the team and it is possible that in case of increased resistance, the rescuer used less force. On the other hand, this may correspond to the clinical situation.

In this experiment, we chose the prearrest recording as the reference section in the EIT analysis for the 30-min compression break recording after realizing that the intra-arrest recordings of our study groups differed too much to provide a reasonable reference point for this more uniform recording point. The distribution of ventilation seemed more dorsally distributed, especially in the CCC group, compared to our previous study. Part of this is probably due to the accumulation of pneumothoraces in the CCC group because the formation of a pneumothorax shifts the distribution dorsally.

The capability of the EIT to produce spatial discrimination was probably reduced due to the untypical tidal ventilatory pattern of two sequential ventilations with a long pause during compressions with the 30:2 protocol. We explored the effect of insufflating the lungs and clamping the endotracheal tube at the end of experiment on the findings in CT scans by re-insufflating the lungs after the first scans and performing a second CT scan round. With some subjects, the atelectatic lung regions differed significantly between these two rounds, depicting the still dynamic state of lung aeration post-mortem and possibly reducing the discriminatory power.

## Conclusions

Our study suggests no difference in arterial blood levels of oxygen, carbon dioxide or lactate, haemodynamics or the distribution of ventilation with compression-to-ventilation ratios of 30:2 compared to CCC during prolonged CPR with 10 cmH_2_O PEEP.

### Supplementary Information

**Additional file 1.** The Arrive Checklist and notations of adherence concerning the manuscript.

**Additional file 2.** The reconstruction options used to process the raw global change in impedance data.

**Additional file 3.** An illustrative image of the workflow used to obtain the mean and regional HU values within one lung CT slice. The lungs have been delineated. The mean HU values of each hemithorax and the accessory lobe are multiplied the according area and the sum of these values divided by the sum of the areas to obtain the mean HU value of the lung tissue in the whole CT slice. The regional mean HU values are obtained as the sum of HU values of the according region divided by the amount of summed regions.

**Additional file 4.** The exploratory analyses performed using the current dataset and the prior dataset of our group [13].

## Data Availability

The datasets used and/or analysed during the current study are available from the corresponding author on reasonable request.

## Abbreviations

ALS: Advanced life support
CCC: Continuous chest compressions
CPR: Cardiopulmonary resuscitation
CT: Computed tomography
DO_2_: Oxygen delivery to tissues
ECPR: Extracorporeal cardiopulmonary resuscitation
EIT: Electrical impedance tomography
EtCO_2_: End-tidal carbon dioxide
FiO_2_: Fraction of inspired oxygen
HU: Hounsfield unit
IQR: Interquartile range
MAP: Mean arterial pressure
NIRS: Near-infrared spectroscopy
OHCA: Out-of-hospital cardiac arrest
PaCO_2_: Partial pressure of carbon dioxide
PaO_2_: Partial pressure of oxygen
PEEP: Positive end-expiratory pressure
ROSC: Return of spontaneous circulation
V/D: Ventral to dorsal
VF: Ventricular fibrillation
