# Supplementary figures and images for: Oxygenation and ventilation during prolonged experimental cardiopulmonary resuscitation with either continuous or 30:2 compression-to-ventilation ratios together with 10 cmH20 positive end-expiratory pressure
Source: Intensive Care Med Exp. 2024 Apr 12;12:36. doi: 10.1186/s40635-024-00620-z (PMC11014827; doi:10.1186/s40635-024-00620-z)

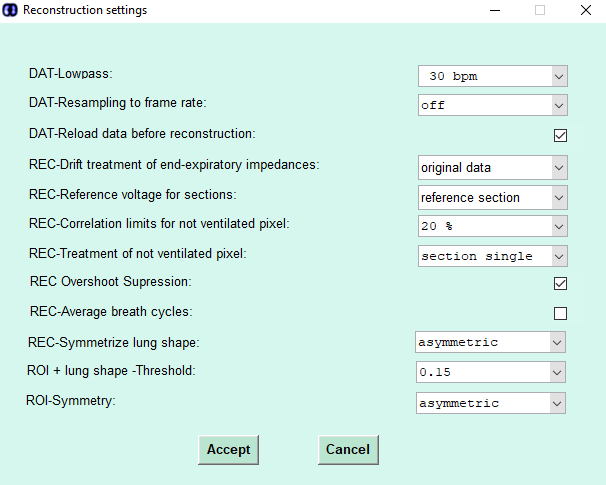

Supplement: Supplementary file 2 — Additional file 2. The reconstruction options used to process the raw global change in impedance data. [file 40635_2024_620_MOESM2_ESM.tiff]

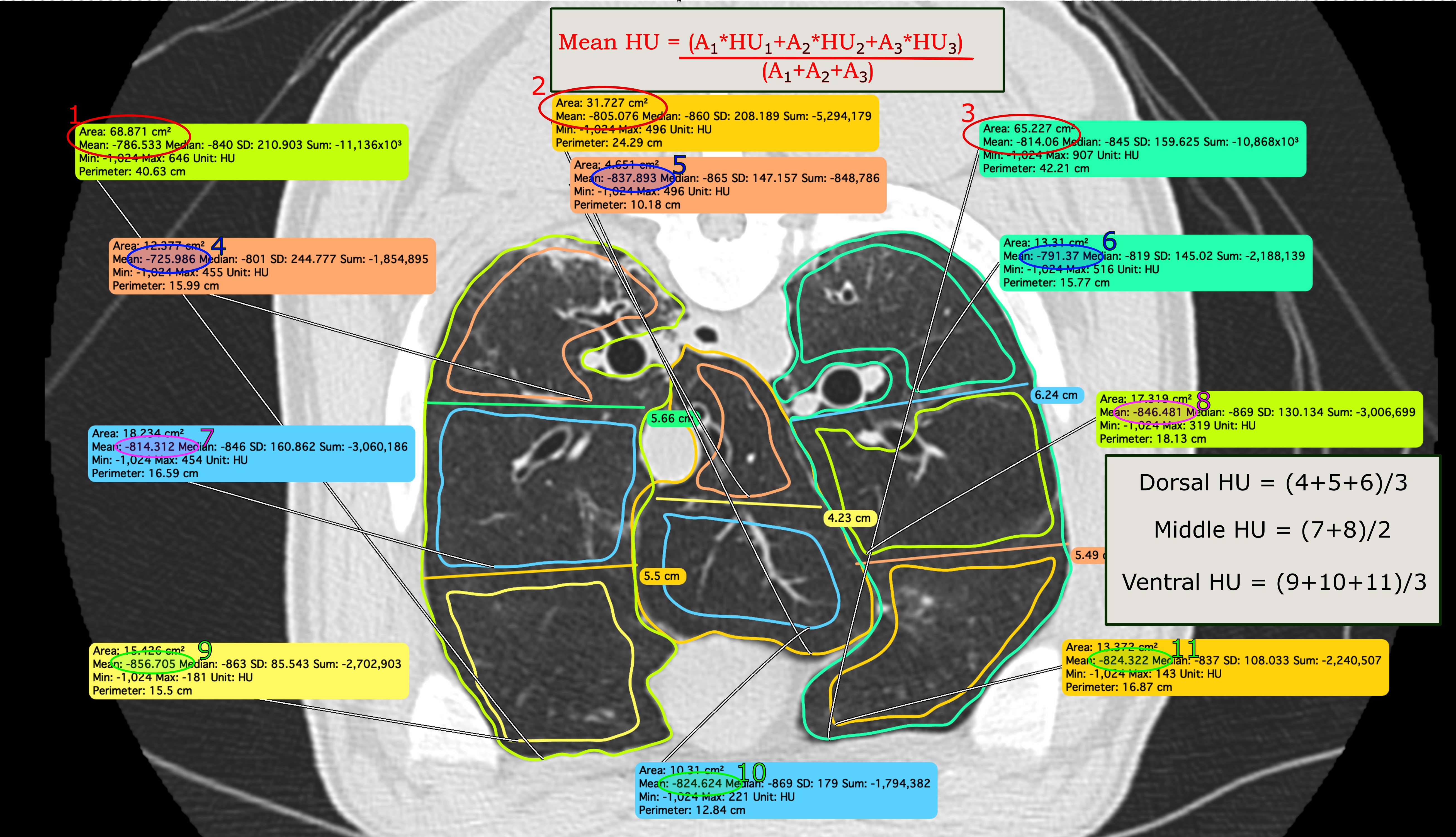

Supplement: Supplementary file 3 — Additional file 3. An illustrative image of the workflow used to obtain the mean and regional HU values within one lung CT slice. The lungs have been delineated. The mean HU values of each hemithorax and the accessory lobe are multiplied the according area and the sum of these values divided by the sum of the areas to obtain the mean HU value of the lung tissue in the whole CT slice. The regional mean HU values are obtained as the sum of HU values of the according region divided by the amount of summed regions. [file 40635_2024_620_MOESM3_ESM.jpg]
