# Supplementary material for: Oxygenation and ventilation during prolonged experimental cardiopulmonary resuscitation with either continuous or 30:2 compression-to-ventilation ratios together with 10 cmH20 positive end-expiratory pressure
Source: Intensive Care Med Exp. 2024 Apr 12;12:36. doi: 10.1186/s40635-024-00620-z (PMC11014827; doi:10.1186/s40635-024-00620-z)
Supplement: Supplementary file 4 — Additional file 4. The exploratory analyses performed using the current dataset and the prior dataset of our group [13]. [file 40635_2024_620_MOESM4_ESM.docx]

**Figure S1**: Arterial oxygen pressures (PaO_2_), carbon dioxide pressures (PaCO_2_), mean arterial pressures, EtCO_2_ and lactate levels during experimental cardiopulmonary resuscitation shown as medians and interquartile ranges. These comparisons have been made between the nonrandomized groups of subjects with PEEP = 0 cmH_2_O (n = 30) and subjects with PEEP = 10 cmH_2_O (n = 31) The p-value is given for a mixed effects model between the groups. P values for the interaction of time x group and F values are given in **Table S1**.

**Table S1**: P values and F values for the PEEP 10 cmH_2_O and PEEP 0 cmH_2_O comparisons. Significant P values are bolded.

|  | **pO_2_** | **pCO_2_** | **MAP** | **EtCO_2_** | **Lactate** |
| --- | --- | --- | --- | --- | --- |
| **Group P value** | **0.032** | **0.018** | 0.078 | 0.58 | 0.92 |
| **Group F** | 4.82 | 5.95 | 3.22 | 0.317 | 0.00943 |
| **Time x Group P value** | 0.082 | **0.0046** | 0.67 | **0.026** | 0.99 |
| **Time x Group F** | 1.82 | 2.98 | 0.708 | 2.31 | 0.201 |

**Figure S2**: paO_2_ pressure comparisons between the nonrandomized groups of 30:2 PEEP 10 cmH_2_O (n = 16), 30:2 PEEP 0 cmH_2_O, CCC PEEP 0 cmH_2_O and CCC PEEP 10 H_2_O during experimental cardiopulmonary resuscitation shown as medians and interquartile ranges. The p-value is given for a mixed effects model between the groups.

The P values for interaction terms of Time x group are: **0.019** (F = 2.36) for 30:2 PEEP 10 vs 30:2 PEEP 0; 0.41 (F = 1.03) for CCC PEEP 10 vs CCC PEEP 0; **0.033** (F =2.14) for 30:2 PEEP 10 vs CCC PEEP 0.
